# The Safety of Bacteriophages in Treatment of Diseases Caused by Multidrug-Resistant Bacteria

**DOI:** 10.3390/ph16101347

**Published:** 2023-09-24

**Authors:** Ka Mun Chung, Sue C. Nang, Swee Seong Tang

**Affiliations:** 1 Division of Microbiology and Molecular Genetics, Institute of Biological Sciences, Faculty of Sciences, University of Malaya, Kuala Lumpur 50603, Malaysia; 2 Department of Microbiology, Biomedicine Discovery Institute, Monash University, Clayton, VIC 3800, Australia; 3 Centre for Research in Biotechnology for Agriculture, University of Malaya, Kuala Lumpur 50603, Malaysia

**Keywords:** human health, phage safety, endotoxin, phage-resistant bacteria, antibiotic-resistant bacteria, animal studies, clinical trials

## Abstract

Given the urgency due to the rapid emergence of multidrug-resistant (MDR) bacteria, bacteriophages (phages), which are viruses that specifically target and kill bacteria, are rising as a potential alternative to antibiotics. In recent years, researchers have begun to elucidate the safety aspects of phage therapy with the aim of ensuring safe and effective clinical applications. While phage therapy has generally been demonstrated to be safe and tolerable among animals and humans, the current research on phage safety monitoring lacks sufficient and consistent data. This emphasizes the critical need for a standardized phage safety assessment to ensure a more reliable evaluation of its safety profile. Therefore, this review aims to bridge the knowledge gap concerning phage safety for treating MDR bacterial infections by covering various aspects involving phage applications, including phage preparation, administration, and the implications for human health and the environment.

## 1. Introduction

The re-emergence of bacteriophages (phages) for the treatment of chronic or difficult-to-treat infections and diseases has been extensively observed in recent years. Phages are viruses that specifically target and kill host bacteria. They serve as a possible alternative to antibiotics in the treatment of bacterial infections, especially in this era where there is a lack of new and effective antibiotics to combat the rise of multidrug-resistant (MDR) bacteria. The term “MDR” refers to organisms that are resistant to at least one antibiotic agent from three or more antibiotic classes [1]. Extensively drug-resistant (XDR) and pan-drug-resistant (PDR) bacteria are also used to categorize bacterial resistance, of which XDR bacteria are non-susceptible to at least one agent in all but two or fewer antibiotic categories, and PDR bacteria are resistant to an agent from all antibiotic classes [2].

The development of phage therapy as an alternative to traditional antibiotics has gained substantial interest over the years. However, its ultimate success in clinical use relies on evidence that proves phage therapy is safe and non-toxic for humans. A major safety concern is the disruption of the body’s microbiome, which is a key regulator of human health. Another concern is the impact of phage on the immune system, which may ultimately lead to chronic inflammation and other immune reactions. As phages are prepared by co-culturing with bacteria, it is crucial to consider the potential risk of endotoxin contamination that could trigger the inflammatory cytokine response, resulting in serious health issues such as toxic shock [3]. Additionally, the use of temperate phages may produce bacterial lysogens that are resistant to the same phage type, potentially increasing their virulence [4].

Recent studies have shown that phages are generally safe and do not produce any adverse effects when used in animals or humans [5,6,7,8]. Nevertheless, a few studies have reported transient adverse events or side effects during phage therapy, which include inflammation, flushing, hypotension, and fever [9,10,11]. The absence of a standard protocol to evaluate the safe usage and preparation of phages results in a lack of consistent, complete, and reliable data to conclude the safety aspect of phage therapy. Hence, a rigorous and detailed exploration of phage safety is required to guide treatment decisions.

In this review, we discuss the advancement, importance, and current applications of phages. Phage applications in clinical settings, the food and agricultural sector (e.g., prevention), and environmental control (e.g., biosensors) are reviewed. It also focuses on the safety concerns and challenges of translating phages from the bench to clinical bedside applications. The topics that are included are the disruption of the microbiome, immunological responses, induction of phage resistance in bacteria, lysogeny, and contaminants (e.g., endotoxins) associated with phage preparation. The environmental safety issue related to phage release is also briefly discussed.

## 2. The Era of Phages

### 2.1. An Alternative to Antibiotic

Ever since the discovery of the “wonder drug” penicillin, antibiotics have been the first line of defense against bacterial infections. Indeed, virtually everyone alive today has grown up in the antibiotic era. However, the overuse of antibiotics has inadvertently led to the emergence of MDR bacteria. For example, infections caused by *Acinetobacter baumannii* such as pneumonia, meningitis, and sepsis have traditionally been treated with beta-lactams [12,13]. However, MDR *A. baumannii* strains, including those resistant to beta-lactams, are increasingly reported; hence, last-line antibiotics such as polymyxins are prescribed for their treatment [14,15]. Thus, *A. baumannii* is listed by the World Health Organization (WHO) as a priority pathogen for which new antibiotics are urgently needed, along with other bacteria such as *Enterococcus faecium*, *Staphylococcus aureus*, *Klebsiella pneumoniae,* and *Pseudomonas aeruginosa*, collectively referred to as the ‘ESKAPE’ pathogens [16]. As MDR bacteria become more prevalent among human populations, they pose a serious threat to clinical and public health, thus becoming a major global health issue. With only a handful of new antibiotics on the horizon [4], there is a growing need to explore alternative antimicrobial strategies, of which phage therapy has become increasingly apparent.

Phages are the most abundant and diverse biological entities that are present on Earth [17]. Phages are highly specific, and they are loosely categorized into monovalent or polyvalent phages depending on their host range. Monovalent phages are defined as phages that have a narrow host range spectrum specific to a single bacterial genus, whereas polyvalent phages are phages that have a broad host range specific to more than two genera [18]. For example, FAHEc1 is a monovalent phage that only targets *Escherichia coli* and can be used to reduce contamination in beef, whereas S5 and vB_EcoM_swi3 are polyvalent and target both *Salmonella* and *E. coli* [19,20,21]. Phages exhibit different life cycles, including the lytic cycle, chronic cycle, lysogenic cycle, and pseudolysogenic cycle [22]. Worth noting, lytic phages are often favored for phage therapy due to their ability to effectively lyse bacteria [15].

### 2.2. Current Phage Applications

Ever since the discovery of phages, researchers have taken advantage of their abundance and infection specificity to combat pathogenic bacteria, mainly for treating bacterial infections, preventing food contamination, and for environmental control. With the emergence of MDR bacteria, research examining phages as an alternative to antibiotics has once again begun in earnest, with the current application of phages against these “superbugs” briefly discussed below.

#### 2.2.1. Phage Therapy against MDR Pathogens

When phages were first identified and described in the early 20th century, they were used mainly for therapeutic purposes against infections such as dysentery, diarrhea, furunculosis (hair follicle abscess), urinary tract infections, and respiratory tract infections. For example, d’ Herelle utilized phage therapy in the treatment of severe dysentery in children (1919) and cholera in India (1931) [23,24,25]. However, the efficacy of these treatments was unclear at the time.

There has been an increasing focus on phage therapy in recent years due to the alarming increase of MDR bacteria. For example, phages AP025 and AP006 showed good infectivity rates when used against 51 clinical strains of MDR *P. aeruginosa* [26]. Furthermore, phages SHWT1 and vB_SalS_TU03 were both shown to target MDR clinical isolates of *Salmonella* spp., suggesting they might be potential therapeutic agents for the treatment of salmonellosis [27,28].

The good efficacy of phage therapy has also been illustrated using in vivo models. Utilizing a mouse model, phages have been found to be effective in eradicating MDR *A. baumannii*, which is known to cause pneumonia, urinary tract infection (UTI), sepsis, and wound infection [8,29]. Phages have also been used in clinical studies and shown to be effective for the treatment of patients with disseminated MDR *A. baumannii* infection [30], MDR *P. aeruginosa* lung infection [31], MDR *K. pneumoniae* post-heart transplant infection [32], carbapenem-resistant *A. baumannii* ventilator-associated pneumonia in COVID-19 [33], and *Mycobacteroides abscessus* chronic lung disease [34].

#### 2.2.2. Food Safety

Since foods of animal origin are frequently contaminated with MDR pathogens, MDR bacterial infections are no longer only associated with hospital-acquired infections and have started to pose a risk to the general public. This is said to be due to the excessive use of antibiotics in feedstock and food preparation [35,36]. MDR strains of *Campylobacter*, *Salmonella*, *Escherichia*, and *Listeria* that are capable of causing disease when ingested have been found in water [37,38,39], meat [40,41,42], vegetables and fruits [43,44,45], and dairy products [46].

Phages can be applied for pre-harvest and post-harvest pathogen control. Studies have been conducted to evaluate the effectiveness of phage therapy against MDR bacteria in livestock. Naghizadeh et al. (2019) [47] demonstrated that TM1 and TM3 phages are capable of clearing colibacillosis in broiler chickens. Phages were also found to effectively treat cattle and swine infected with Shiga-toxigenic *E. coli* [48,49], methicillin-resistant *S. aureus* (MRSA) [50], and MDR *Salmonella* strains [51]. In aquaculture, phages are being applied to seafood, such as oysters and farmed shrimp, as a biocontrol agent against *Vibrio* spp., which are known to easily gain resistance against antibiotics [52,53]. Phages are also often used in post-harvest control for preservation. Phages are added to tomatoes [54], potatoes [55], onions [56], and German turnips [57] to prevent soft rot disease and enhance the crops’ lifespan and freshness. Several phage products have also been approved by the Food and Drug Administration (FDA). Examples of these products are EcoShield PX™, which targets Shiga toxin-producing *E. coli* in meat products, and ShigaShield™, which is designed to treat foods with a high risk of *Shigella* contamination, such as meat, poultry, seafood, vegetables, and dairy [58,59]. Overall, phages play an important role in preventing zoonotic pathogens from being transferred to humans.

#### 2.2.3. Environment Pathogen Control

Bacteria are capable of surviving in the environment, including soil. These bacteria often thrive in soil supplied with insecticide or decomposed organic matter. Importantly, some of these bacteria are reported to exhibit the MDR phenotype. For example, *Bacillus* spp. isolated from soil exposed to insecticides were found to be resistant to chloramphenicol, ampicillin, cefotaxime, and streptomycin [60]. In addition, Mahdiyah et al. (2020) [61] isolated MDR bacteria, including extended-spectrum beta-lactamase (ESBL)-producing *E. coli* and MRSA, from peat soil. Recent research has also found that the soil microbial community is able to disseminate antimicrobial resistance through horizontal gene transfer, hence contributing to the abundance of MDR bacteria in the environment [62].

Phages are able to act as bioindicators to detect the presence of MDR bacteria in the environment as well as biocontrol agents. As feces are often a source of MDR bacterial infection (e.g., cholera, dysentery, and food poisoning), early warning of sewage contamination can be detected by phage fecal indicators. Yahya et al. (2015) [63] demonstrated that the most abundant indicator phages in samples collected from three water treatment plants were somatic coliphages, followed by F-specific RNA phages and Bacteroides-infecting phages. As a bioindicator tool, phages can be attached to a sensor surface to detect pathogens in samples [4]. Phages can also be used to control MDR bacteria in soil. For example, in two studies, the concentration of both MDR bacteria and antibiotic-resistant genes in the soil decreased by 2–6-fold following phage treatment [64,65]. Therefore, applying phage therapy to the environment may decrease the further spread of MDR bacteria to animals and humans.

## 3. Safety Concerns and Challenges

Despite successful cases of treatment with phage therapy, the safety and potential side effects pose a significant challenge to its broader application, especially for clinical use. The concerns associated with the human body include the impact of phage on the microbiome, lysis-induced endotoxin release, immune activation, and increased bacterial resistance. Phage usage may also have environmental implications.

### 3.1. Human Body

#### 3.1.1. Disruption of Gut Microbiome and Host Genome

Many broad-spectrum antibiotics are prone to inducing superinfections such as MDR *Candida albicans* yeast infections and *Clostridium difficile* colitis [66]. In contrast, phages are thought to have only a minimal impact on the body’s normal flora [66]. The gut microbiota is becoming more widely acknowledged as a key regulator of human health. Several studies have confirmed that phages are able to eliminate the target bacterial organism in feces without any considerable alterations to the gut microbiota. For example, oral consumption of two phage cocktails curated by McCallin et al. (2013) [67] and Febvre et al. (2019) [68] reduced the target organisms and was associated with no evidence of adverse events. Phage therapy has also been shown to have a lower impact on the gut microbiota than antibiotics. For example, in a murine model, phages reduced the gut carriage of MDR uropathogenic *E. coli* (UPEC) with minimal microbiome impact compared with antibiotic treatment. After receiving antibiotics (5 mg/mL streptomycin sulfate and 1 mg/mL kanamycin sulfate), 50 gut microbiome genera were reduced and 14 had increased, whereas with phage therapy, only 11 species were observed to have reduced in abundance while 21 had increased [69]. On the contrary, phages have also been used as potential microbial modifiers in gastrointestinal diseases caused by infection and an unbalanced microbiome [70].

Careful consideration is essential when determining phages for therapeutic use, as certain phages have the ability to transmit genes to bacteria, particularly antibiotic resistance genes (ARGs) [71]. ARGs on bacterial chromosomes or plasmids may occasionally be integrated into the phage capsid or DNA during the assembly of phage components to create progeny [72]. As these progeny phages are released, they can re-infect hosts to disseminate these ARGs [73]. There is a large community of phages in the human gut and other environments that are ARG carriers. Antibiotic use has resulted in an increase in the number of these ARG-carrying phages in the human gut [74]. It is evident that transduction is a driving force behind the genetic diversity in *E. coli* strains that colonize the gut, contributing to the dissemination of drug resistance among gut bacteria [75]. However, there is currently a lack of knowledge to inform the extent to which phages play a role in the horizontal transfer of ARGs. One study utilized a combined approach of bioinformatics and experimental analysis to access ARGs in phage genomes [76]. It was reported that the ARG abundances in 1181 phage genomes were vastly overestimated using exploratory thresholds due to the matching of proteins that are unrelated to antibiotic resistance. Four predicted ARGs were evaluated experimentally and were not found to confer antibiotic resistance in *E. coli*. In-depth investigation is required to evaluate the impact of phage-mediated horizontal ARG transfer on human health.

Phage cocktails (i.e., combinations of phages) have been commonly employed to increase the efficiency and/or broaden the coverage of targeted bacteria. The increased number of phages used may potentially affect non-targeted bacteria, even though the impact may not be as significant as antibiotics [77].

#### 3.1.2. Bacterial Endotoxin Release

Given that effective phages can lyse bacteria within minutes of commencing therapy [11], phage therapy may induce a rapid and significant release of endotoxins. This has been demonstrated when treating MDR uropathogenic *P. aeruginosa* [78] and represents a serious health issue given that endotoxin is one of the most potent triggers of the inflammatory cytokine response [3]. Cytokines cause symptoms such as flushing, irregular heart rate and blood pressure, dyspnea, fever, urticaria, edema, nausea, and rashes [79]. When the condition worsens, an inflammatory cascade can eventually result in multiple organ failure [80]. The limited data on endotoxin release during phage therapy is hindering a comprehensive understanding of the potential effects of endotoxin responses on human health.

#### 3.1.3. Impact of Phages on Immune Activation

Phages may stimulate both innate immunity (by triggering phagocytosis and cytokine production) and adaptive immunity (by influencing the synthesis of antibodies and effector polarization). Utilizing in vitro cell culture models, *S. aureus* phages were found to suppress lipopolysaccharide (LPS)-induced inflammation in mammalian epithelial cells and induce pro- and anti-inflammatory responses in peripheral blood mononuclear cells [81,82]. In in vivo studies, phages have been shown to promote the production of phage-specific antibodies and induce slight inflammation in mice [83,84]. On the contrary, Dufour et al. (2019) [83] found that phage treatment led to a reduction in inflammation and a quicker normalization of aberrant blood cell counts compared with antibiotics.

There are conflicting reports on phage-induced antibody production. While an increase in the production of IgG and IgM was reported following intraperitoneal administration of phages for treatment of MDR *V. parahaemolyticus* infection and *S. aureus* bacteremia [85,86], some clinical studies conducted on healthy adults and children diagnosed with acute bacterial diarrhea have shown no increase in the titers of IgG, IgM, IgA, and sIgA following phage administration [31,78,87]. Although severe immune reactions related to high-dose phage use have not been reported, it is always advisable to not administer a high dosage of phage to avoid potential severe immune reactions [88]. Attributing these immune-associated safety concerns solely to phages can be challenging, as other factors, such as poor phage purification, can contribute to the adverse effects [89].

#### 3.1.4. Bacterial and Toxin Contaminants

Apart from the phage’s inherent characteristics, the safety of the phage product can be influenced by the presence of bacterial and chemical contamination during phage preparation.

During phage amplification, a large amount of endotoxins is released as the bacteria undergo lysis. These endotoxins may be difficult to remove completely via purification and may induce infusion-related reactions [81]. Thus, the endotoxin content of a phage product must be maintained within the acceptable range set by the Food and Drug Administration (FDA) for the product to be considered safe for application. These limits vary depending on the route of administration. The upper limit of bacterial endotoxin concentration in phage preparation for subcutaneous injections is 0.5 endotoxin units (EU)/mL, while for intravenous (IV) and intrathecal (IA) injections it is 5 EU/kg of body weight/hour and 5 EU/kg, respectively [90,91]. For in vivo applications, Bao et al. (2020) have proposed an acceptable endotoxin level of less than 1 EU/mL [92]. Most phages prepared in recent clinical studies have bacterial endotoxin levels below the acceptable FDA value [10,30,83,93]. However, some studies have shown that dilution is required to attain endotoxin levels that meet clinical limits [94]. A standardized protocol to effectively remove endotoxin for phage preparation should be made available to ensure that a proper procedure can be widely adapted.

Other than endotoxins, contaminants such as bacterial DNA may also be present in phage preparation and hinder therapy results. Generally, the insertion of bacterial DNA into the human genome may work as a *cis*-element to modify host gene activity, activate proto-oncogenes, silence tumor suppressor genes, and control pathways involved in cancer [95]. However, it is unclear whether this contaminant would influence phage safety, as most case reports and clinical studies do not report on the presence of host bacterial DNA as part of the safety assessment. Only a small subset of studies, such as Gilbey et al. (2019) [96] and McCallin et al. (2013), have reported the absence of bacterial DNA in the phage products used in their studies [67].

Another potential contaminant during the preparation of phages is bacterial toxin. Staphylococcal enterotoxin B is a potent bacterial superantigen capable of causing significant inflammatory responses and potentially being lethal at an LD_50_ of 20 ug/kg and an ED_50_ of 400 ng/kg [97,98]. Although it is doubtful that high enterotoxin doses would be administered during phage therapy, it is also unknown what the appropriate level of enterotoxin is, particularly when administered repeatedly. Other contaminants that might cause safety issues include exotoxins such as alpha hemolysin [99] and lipoteichoic acid [100]. *S. aureus* is known to secrete alpha hemolysin, which would generally cause pneumonia, skin and soft tissue infections, sepsis, arthritis, and abscess formation [101]. Lipoteichoic acid, present in Gram-positive bacteria, may induce fever [102].

Phage purification traditionally involves polyethylene glycol (PEG)-based precipitation, followed by caesium chloride (CsCl) density gradient centrifugation and a final dialysis step to remove CsCl. CsCl is highly toxic to cells when present in high concentration, causing symptoms such as hypotension, numbness, and gastrointestinal discomfort. However, it was said that the residual CsCl levels in the phage products would typically be insignificant [103]. Over the past year, alternative purification methods have been described, including the use of 1-octanol extraction and anion exchange chromatography for the removal of endotoxins [104].

### 3.2. Temperate Phages

Lytic phages are unanimously preferred over temperate phages for therapy purposes. The administration of temperate phages may not be favored due to their intrinsic nature, which favors the lysogenic cycle, in which their genomes are integrated into the bacterial genome and do not lead to immediate bacterial killing. Importantly, bacterial lysogens often exhibit resistance to subsequent infections by phages [4]. Superinfection exclusion and superinfection immunity are the mechanisms that prevent superinfecting phages from either entering the bacterial cell or blocking their infection cycle within the bacterial cell [66].

Temperate phages have the ability to obtain ARGs and virulence genes from the infected host and subsequently transfer them to other bacterial hosts. This process can inevitably lead to the widespread distribution of these factors among bacteria, contributing to the development of difficult-to-treat MDR bacteria. Hence, it is advisable to refrain from using temperate phages in phage treatment until more comprehensive research is available to guide their application. In fact, researchers have been exploring phage engineering techniques to modify temperate phages, making them safe and effective for use in phage therapy.

### 3.3. Induction of Phage-Resistant Bacteria

**Figure 1:**
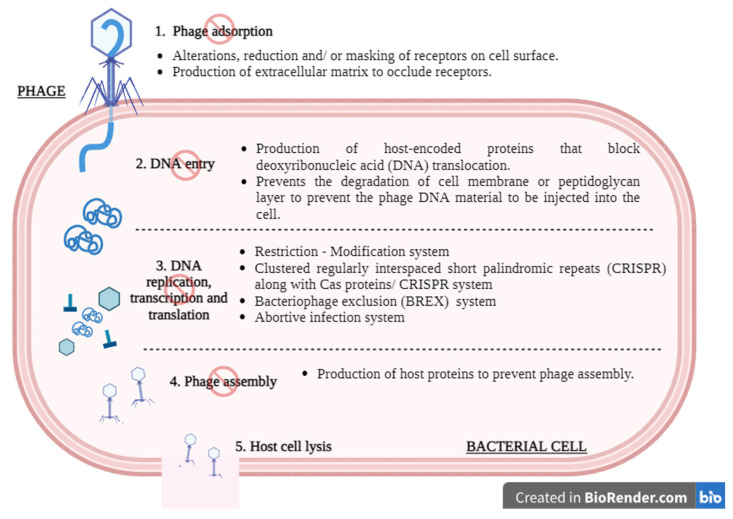
A schematic illustration of the mechanism of phage resistance in relation to the general lytic phage cycle. (1) Phages can attach to the surface of host bacteria using receptor-binding proteins (RBPs). Bacterial cells have various ways to disrupt this attachment process, such as by alternating, reducing, and/or masking their receptors on the surface of the cell. (2) During translocation, phage DNA is directly injected into the host cell’s cytoplasm. Certain bacterial strains can hinder this injection by employing proteins encoded by the host that interfere with the transfer of phage nucleic acid. (3) Once inside the host cytoplasm, phage DNA undergoes preparation for replication. Whether the phage DNA is modified or unmodified, it can be degraded by host-encoded proteins following its translocation into the host cytoplasm. Normally, replication, transcription, and translation of the phage DNA will proceed as usual. However, bacterial cells may be able to resist this action through the initiation of the Restriction–Modification system, the CRISPR system, the BREX system, or the abortive infection system. (4) Assembly and packaging are crucial steps in the production of new phage particles. Certain bacterial strains express proteins that interfere with the assembly or packaging process of the newly synthesized virions. These proteins disrupt the proper formation of the phage capsid or the packaging of phage DNA into the capsid. (5) If the lytic phages manage to pass through all the bacterial defenses, it will eventually lead to host cell lysis. Created with Biorender.com.

As phages are increasingly employed for the treatment of bacterial infections, it has unavoidably led to the emergence of phage-resistant bacteria. It has been reported that bacteria can develop phage resistance through various mechanisms, which include receptor alterations, abortive infection systems, bacteriophage exclusion (BREX), and quorum sensing defense [105]. An example of receptor alteration is observed in *Bordetella* spp. that prevents infection from phage BPP-1 by suppressing the expression of the phage receptor pertactin autotransporter (Prn) [106]. The abortive infection (Abi) system is a strategy that induces host cell death, thus limiting phage propagation. An example of Abi involves the toxin-antitoxin systems, of which *Erwinia carotovora* subspecies *atrosepticawa* was found to consist of a *toxI* gene that encodes for an antitoxin that neutralizes ToxN [107,108]. The BREX defense system is another mechanism that has been increasingly reported. *Bacillus cereus* consists of six-gene cassettes called BREX defense systems that are subject to substantial horizontal gene transfer and offer total phage resistance to a variety of phages, including lytic and temperate phages [105]. A summary of the mechanism of phage resistance in relation to the life cycle of phages is presented in Figure 1.

### 3.4. Environmental Impact

The application of phage therapy may directly or indirectly affect ecosystem biodiversity as phages are released into the environment; however, there is limited research on the environmental impact.

Research has been conducted to compare the disposal of therapeutic phages and broad-spectrum chemical antibiotics in the environment. There has been an increasing focus on antibiotic surveillance and investigations into strategies for appropriate antibiotic disposal [109]. The presence of antibiotic traces in the environment, such as surface and ocean waters, has resulted in increasing public concern due to the potential for promoting the emergence of MDR bacteria. Contrary to antibiotics, discarded therapeutic phages are said to only have a minimal effect on a small group of bacteria as they are often originally isolated from the environment. Furthermore, phages can be rapidly inactivated if they are not adapted to harsh environmental factors, such as extreme temperature, humidity, and UV light [66]. Worth mentioning is that phages are able to withstand a wide range of temperatures (generally 40–70 °C) [110], therefore making them more thermally stable in comparison to antibiotics.

Although phages are naturally present in the environment, the release of high phage concentrations as a result of phage therapy could potentially cause an imbalance in the ecosystem by disrupting the natural microbial balance [111]. Hence, proper waste management represents an essential area that should be put in place to protect the environment and safeguard public health.

## 4. Studies on Phage Safety

Several animal studies [6,8,83], case reports [5,7,10], and clinical trials [13] have been conducted to monitor the safety of phages in the treatment of MDR bacteria.

### 4.1. Animal Studies

Murine and sheep are often used as animal models for studying phage safety. The use of the sheep model provides an advantage over the more commonly used murine model as it is one of the most significant representations of human organ systems, providing a more accurate assessment of phage safety in humans.

Phage therapy has been demonstrated to be safe and effective in treating pathogenic *E. coli*, *P. aeruginosa*, *S. aureus,* and *A. baumannii* in sheep and mouse models [6,83]. Utilizing a murine model, phage 536_P1 successfully eradicated *E. coli* infection without inducing an innate inflammatory response [83]. In another study conducted by Yin et al. (2017), phage Abp1 was effective in eliminating MDR *A. baumannii* in mice without eliciting any cytotoxic effects [8]. Employing a sheep rhinosinusitis model, the administration of a phage cocktail locally at the sinus for treating *P. aeruginosa* infection resulted in no significant adverse effects, such as loss of appetite, fever, or other signs of systemic illness [6]. Similarly, the treatment of *S. aureus*-associated rhinosinusitis in sheep with the phage cocktail NOV012 was not associated with tissue damage or inflammatory infiltration [112]. Details for these animal studies are summarized in Table 1.

### 4.2. Clinical Cases

Multiple case studies have included phage safety monitoring. Liu et al. (2021) have conducted one of the first systematic literature reviews on phage therapy clinical cases where phage safety monitoring alongside treatment is included [113]. These case studies mostly involve patients with conditions such as cystic fibrosis, prosthetic knee infections (PKI), urinary tract infections (UTI), surgery/transplant-related wound infections, and abscesses.

As endotoxins are a major source of phage contamination that may lead to adverse effects during therapy, several case reports have reported the endotoxin level of the phage products used for clinical treatment. While most of the studies reported endotoxin levels within the FDA limits [10,114,115], dilutions were required by some phage products to achieve safe endotoxin levels [11,30,116]. The process of diluting phages to adhere to clinical endotoxin limits results in a reduced phage concentration, potentially compromising the effectiveness of therapy [94]. Due to the lack of standardization, some studies do not include this safety aspect in their research, and consequently, endotoxin levels are not reported [5,117].

In one study, inflammatory changes were assessed following IV and/or intraarticular (IA) administration of SaGR51Φ1, along with antibiotic administration. This study reported no significant inflammatory effect except for a transient, reversible transaminitis [10]. Most cases have reported little to no significant adverse reactions or that the effects were not phage-related (Table 2). Of note, transient fever represents the most associated adverse effect reported; however, fever is a common physiological response to infection.

Overall, it is crucial that standardized therapeutic guidelines are made available to promote consistency and uniformity in monitoring the safety profile of phage therapy, enabling a more reliable conclusion to be drawn.

### 4.3. Clinical Trials

Clinical studies that evaluate the safety aspects of phage therapy are scarce. In a clinical trial to test the efficacy and safety of phage therapy for treating urinary tract infections caused by MDR uropathogens (*Enterococcus* spp., *E. coli*, *Proteus mirabilis*, *P. aeruginosa*, *Staphylococcus* spp., and *Streptococcus* spp.), patients were subjected to either receiving Pyo phage (intravesically), placebo, or antibiotics [134]. The lowest occurrence of adverse effects was demonstrated by the phage group (21%; 6/28) in comparison to the placebo (41%; 13/32) and antibiotic (30%; 11/37) groups. Another phage therapy clinical trial was conducted on patients with MRSA infections. The safety aspects that were considered in this study include pain and systemic adverse reactions [135]. When patients were treated with phage AB-SA01, no adverse events were reported, although there was a notable decrease in inflammation markers and an increase in cytokine interactions. In a clinical trial involving the treatment of chronic ear infections caused by antibiotic-resistant *P. aeruginosa*, Biophage-PA significantly reduced the bacterial load, and no phage-related adverse effects were observed [136]. The details of these clinical trials are shown in Table 3. The presented clinical studies were chosen based on the criteria that they are recent studies involving phage therapy, specifically related to MDR bacterial infections, and that they evaluate the safety aspect of phage therapy.

## 5. Challenges and Future Improvements

Difficulties arise when attempting to compare the data across published clinical cases and clinical trials, as different studies employ their own set of treatment and monitoring protocols [113]. In order to improve current practices, the establishment of a gold standard or standard operating procedure is of paramount importance to guide phage preparation, storage, and transport, as well as the monitoring criteria for determining the efficacy and safety of phage therapy. The purified phage lysate should also adhere to the Good Manufacturing Practice (GMP) guidelines to ensure safety [129].

When phage products are used for therapeutic purposes, information such as the phage genome, titer, purity, and endotoxin level should be made available. Insufficient and incomplete data regarding phage safety monitoring represents another limitation to fully understanding the safety profile of phage therapy. The safety endpoints that can be included are evaluation of the patient’s physical symptoms, chemical lab assessment (e.g., kidney and liver function tests, electrolytes, and inflammation markers), hematology lab assessment (e.g., complete blood count), and immunological response evaluation (e.g., antibodies) [113]. The availability of this comprehensive data would permit a more thorough safety assessment of phage therapy, thus facilitating policymakers’ efforts to establish a comprehensive regulatory framework for phage treatment [137].

Phages are often used in combination with antibiotics to achieve better antibacterial activity [5,87,117], achieved by phage-antibiotic synergy (PAS) [138]. For instance, Uchiyama et al. (2018) evaluated phage-antibiotic combinations against *P. aeruginosa* and observed that combining piperacillin and ceftazidime with *P. aeruginosa* phage KPP22 showed the strongest PAS [139]. Understanding the mechanisms underlying PAS is the key to designing phage-antibiotic therapy. Utilizing metabolomics, it was revealed that the combination of polymyxin B and phage pK8 caused a prolonged inhibition of the citrate cycle, pentose phosphate pathway, and amino acid and nucleotide metabolism *of K. pneumoniae* [140]. It should also be noted that antagonistic phage-antibiotic combinations are often overlooked. A recent study by Zuo et al. (2021) showed that replication of coliphage T3 was impeded by aminoglycoside antibiotics (e.g., neomycin and kanamycin), which inhibit protein synthesis [141]. Overall, careful consideration and more research are needed to inform appropriate concentrations, application timing, and optimal selection of phages and antibiotics based on patients’ diagnoses and medical histories.

In order to further advance the field of phage therapy, it is essential to establish comprehensive phage libraries consisting of a diverse array of phages that are known to be safe and effective against bacterial infections. By having such accessible repositories of phages, the potential for finding suitable phages can be significantly enhanced.

## 6. Conclusions

Since their discovery, phages have been extensively utilized in the agricultural and environmental sectors to ensure food safety and effective pathogen control. Phages have also re-emerged as an alternative to antibiotics due to the emergence of MDR bacteria. As phage therapy research against MDR bacteria advances quickly, ensuring its safety in clinical applications becomes an absolute priority.

The recent success of in vivo studies, case reports, and clinical trials has demonstrated that phages exhibit a relatively safe profile and are typically tolerable when administered to animals and humans. These encouraging findings solidify the foundation for the broader application of phage therapy as a safe and well-tolerated treatment modality. Given existing knowledge gaps and limited data on the potential health implications of phage therapy, it will be imperative to address and overcome the safety concerns discussed in this paper. It is also vital to establish and implement standardized safety assessments. By adhering to consistent and rigorous safety evaluation protocols, researchers can effectively address any uncertainties and validate the reliability of phage therapy, reinforcing its position as a trustworthy medical approach.

Nonetheless, ongoing research in the phage field enables researchers to enhance their comprehension of the safety aspects surrounding phage therapy for combating MDR bacteria. While there remains a considerable journey before phage therapy becomes an established standard of clinical care, it is crucial to continue expanding the knowledge base and facilitating the translatability of phages from bench-side to clinical bedside applications.

## Figures and Tables

**Table 1:** A compilation of animal studies that have conducted safety monitoring on phage therapy against MDR bacteria.

Studies	Phage/s		Against	Phage Distribution	Normal Imaging/Lab Assessment	Presence of Abnormal (Increase or Decrease) of		Phage-Related Adverse Events (Cytotoxicity or Physiological Effects)
	Phage/s (Administration Route)	Endotoxin within Acceptable Range				Cell Infiltration/Cytokine Production	Antibodies Production	
Dufour et al., 2019 [83] Mice	*E. coli* phages LM33_P1 and 536_P1 (Intranasal)	Yes, 0.072 and 0.003 EU/mL, respectively	Pathogenic *E. coli*		Yes	Not significant		
Fong et al., 2019 [6] Sheep	*P. aeruginosa* phage cocktail (Local)		Chronic rhinosinusitis (CRS) *P. aeruginosa* strain	Detected in feces on Day 7 of treatment Detected in blood samples of certain sheep on Day 1 and Day 7 of treatment Detected in organ samples after 16–18 h of treatment	Yes			No significant adverse effects such as loss of appetite, fever, or other signs of systemic illness
Drilling et al., 2017 [112] Sheep	NOV012 cocktail (Local)		CRS *S. aureus*	Not detected in blood during 20 days of treatment		None		No adverse effects
Yin et al., 2017 [8] Mice	Abp1 phage (Intraperitoneal)	Only mentioned that the endotoxin is removed using a kit	MDR *A. baumannii*	Detected in liver and kidney 7 days after infection				No cytotoxic effect

Gray columns indicate that the respective aspects were not reported in the study. CRS: Chronic rhinosinusitis; MDR: Multidrug resistant.

**Table 2:** A compilation of case studies (2017–2022) that have conducted safety monitoring on phage therapy against MDR bacteria.

Studies	Phage/s		Against	Phage Distribution	Normal Imaging/Lab Assessment (e.g., X-rays, Liver Function)	Presence of Abnormal (Increase or Decrease) of		Phage-Related Adverse Events (Physiological Effects)
	Phage/s (Administration Route)	Endotoxin within Acceptable Range				Cell Infiltration/Cytokine Production	Antibodies Production	
Eskenazi et al., 2022 [118]	Phage vB_KpnM_M1 (M1) (Local)		Fracture-related pandrug-resistant *Klebsiella pneumoniae* infection		Yes		Neutralizing antibodies are triggered	None
Lebeaux et al., 2021 [7]	APC 2.1 cocktail (Inhalation)	Yes, 30 mL of APC 2.1 was diluted tenfold from stock (5 × 10^9^ pfu/mL, 1760 EU/mL endotoxin level)	Pandrug-resistant *Achromobacter xylosoxidans* in lung transplanted cystic fibrosis infection					Initial persisting airway colonization with no adverse effects
Johri et al., 2021 [119]	Pyo, Intesti, and *Staphylococcal* phage (Oral, intrarectal, and urethral instillations)		Methicillin-resistant *Staphylococcus aureus* (MRSA) and *Staphylococcus haemolyticus*, *Enterococcus faecalis*, and *Streptococcus mitis* in chronic bacterial prostatitis		Yes	None	None	None
Wu et al., 2021 [33]	ɸAb124 (Inhalation)		Carbapenem- resistant *A. baumannii* (CRAB) secondary to COVID-19 infection		Yes	Present (Atypical cytokine storm, dramatic increase of IL6 and IL8 during initiation)		Transient fever
Ramirez-Sanchez et al., 2021 [120]	AB-SA01 cocktail and SaGR51ø1 phage (IV and IA)	Yes, <250 EU/mL (<5 EU/kg per dose) and <1 EU/mL, respectively	Persistent methicillin- sensitive *S. aureus* (MSSA) in prosthetic joint infection		Yes	None	Neutralizing antibodies are triggered	Liver function tests, renal function, and complete cell blood count remained stable
Khatami et al., 2021 [115]	PASA16 (IV)	Yes, 170 EU/mL.	MDR *P. aeruginosa*	Detected in blood during initiation (Day 2 and 5)	Yes	Present (CRP peaked)	Present (Increased serum IgG)	Transient fever and transient increase in pain at the infected site (heel)
Ferry et al., 2020 [5]	PP1493, PP1815, and PP1957 phage cocktail (Local)		Orosthetic knee infection (PKI) Methicillin- susceptible *S. aureus*			Present (Mild synovitis)		Mild discharge of synovial fluid and mild synovial inflammation without adverse effects or superinfection
Bao et al., 2020 [117]	Cocktail III composed of Kp152, Kp154, Kp155, Kp164, Kp6377, and HD001 (Local)		Extensively drug-resistant *Klebsiella pneumoniae* in UTI					None
Rostkowska et al., 2020 [121]	Phage therapy (Intrarectal)		MDR ESBL- producing *Klebsiella pneumoniae* in UTI		Yes	None		Yes
Doub et al., 2020 [10]	*S. aureus* phage, SaGR51Φ1 (Intraarticular/Intravenous)	Yes, <1 EU/mL	Chronic methicillin-resistant *S. aureus* prosthetic joint infection		Yes	Present (Transaminitis)		Transient transaminitis
Rubalskii et al., 2020 [32]	Various phage cocktails (Local, Oral, Inhalation)		MDR/especially recalcitrant *S. aureus*, *Enterococcus faecium*, *P. aeruginosa*, *K. pneumoniae*, and *E. coli* in cardiothoracic surgery infection		Yes	Present		None
Aslam et al., 2020 [116]	Patient 8 SDSU1 cocktail, SDSU2 cocktail, and PAK_P1 single phage (IV)	Yes, 4.3 EU per dose. Diluted.	MDR and antibiotic-recalcitrant *P. aeruginosa*					Transient fever, wheezing, and shortness of breath
Gainey et al., 2020 [87]	Ax2CJ45ϕ2 (Intravenous)		Pan-drug resistance *Achromobacter* spp. In cystic fibrosis		Yes			No infusion site reactions, anaphylaxis, elevated liver enzymes, increased serum creatinine, electrolyte abnormalities, seizures, abnormal vitals, and gastro- intestinal disturbances
Corbellino et al., 2020 [122]	vB_KpnM_GF (Oral and Intrarectal)		MDR Carbapenemase-Producing *Klebsiella pneumoniae*		Yes			Normal clinical examinations, complete blood count, the serum C-reactive protein levels, and the concentrations of liver enzymes and serum electrolytes. No adverse effects
Aslam et al., 2019 [31]	Various phage cocktails and single phage (Intravenous/Inhalation)	Yes, between 0.2 EU/mL and 7300 EU/mL	MDR infections caused by *P. aeruginosa* and *Burkholderia dolosa* in lung transplant		Yes	Present		None
Nir-Paz et al., 2019 [123]	ɸAbKT21phi3; MK278859; and ɸKpKT21phi1; MK278861 (Intravenous)	Yes, 35 EU/mL for ɸKpKT21phi1 and 5 EU/mL for ɸAbKT21phi3	Extensively drug-resistant *A. baumannii* and MDR *K. pneumoniae* in bone infection	Not detected in blood, stool, urine, or saliva after 8 months	Yes	None		None
Tkhilaishvili et al., 2019 [124]	Phage (Local)		MDR *P. aeruginosa* in Periprosthetic Joint Infection					None
Onsea et al., 2019 [114]	BFC 1 and Pro phage cocktails (Local)	Yes.	MDR *S. epidermidis*, MDR *P. aeruginosa*, *S. aureus*, *Sagalactiae,* and *E. faecalis* in musculoskeletal infections		Yes	None	None	No severe systemic side effects or immune reactions The systemic inflammatory markers (CRP and WBC count) decreased to normal levels after one month, and no antibodies were produced against the administered phages
Maddocks et al., 2019 [125]	AB-PA01 (Inhalation, IV)		MDR *P. aeruginosa* in ventilator-associated pneumonia and empyema					None
Law et al., 2019 [126]	AB-PA01 (IV)		MDR *P. aeruginosa* in cystic fibrosis		Yes			No adverse events noted clinically or on laboratory monitoring (liver function tests, complete blood counts, electrolytes)
Dedrick et al., 2019 [9]	Three phage cocktail—Muddy, BPs33ΔHTH-HRM10, and ZoeJΔ45 (IV)	Undetectable levels of endotoxin	MDR *Mycobacterium abscessus*	Detected in serum after 1 day of treatment Detected in feces 4 and 6 days post-treatment Detected in wound swabs at 3 and 5 days post-treatment Undetected in saliva, although a high phage titer was observed on Day 9 after treatment initiation	Yes		Present	Transient sweats and flushing for the first 2 days of therapy, but continued therapy without event
Kuipers et al., 2019 [127]	Anti-*Klebsiella pneumoniae* phages (Oral/intravesical)	No exact details of endotoxin concentration	MDR-ESBL-producing *K. pneumoniae* in chronic UTI					None
Aslam et al., 2019 [128]	AB-SA01 cocktail (IV)		MSSA in left ventricular assist device infection					No adverse clinical or laboratory events
Ferry et al., 2018 [129]	*Staphylococcal* phage Sb-1 (Local)	Yes, <1–5 (EU)/mL for 10^10 ^pfu/mL	XDR *P. aeruginosa* in complex bone and joint infection					
Duplessis et al., 2018 [130]	Phage cocktail (IV)	Yes, <5 EU/kg per hour Diluted to meet standard.	MDR *P. aeruginosa* in bacteremia					None
LaVergne et al., 2018 [11]	Phage (IV)	Yes, <5 EU/kg per hour Diluted to meet standard.	MDR *A. baumannii* in craniectomy site infection	Detected in blood during initial administration	Yes			Transient hypotension
Ferry et al., 2018 [131]	Phage cocktail (Local)		MDR *P. aeruginosa* and methicillin- susceptible *S. aureus* in Prosthetic-Joint Infection					None
Ujmajuridze et al., 2018 [78]	Adapted Pyo phage (Intravesical)		MDR uropathogens (*S. aureus*, *E. coli*, *Streptococcus* spp., *P. aeruginosa*, and *Proteus mirabilis*) in UTI					Transient fever and chills
Schooley et al., 2017 [30]	ΦPC, ΦIV, and ΦIVB (IV and Intracavitary)	Yes, 2.4 × 10^3^ endotoxin units (EU)/mL, 5.89 × 10^3^ EU/mL, and 1.64 × 10^3^ EU/mL, respectively. Diluted to meet standard.	MDR *A. baumannii* in infection	No detectable phage titer in plasma samples after 6 h following initial injection	Yes			None
Zhvania et al., 2017 [132]	Sb1, Pyo, and Fersis phages (Local)		Antibiotic-resistant chronic *S. aureus* in skin infection		Yes			None
Jennes et al., 2017 [133]	BFC1 cocktail (IV)		MDR *P. aeruginosa* in acute kidney injury		Yes	None		No unexpected adverse events, clinical abnormalities, or changes in laboratory tests

Gray columns indicate that the respective aspects were not reported in the study. MDR: Multidrug resistant; MRSA: Methicillin resistant *S. aureus*; CRAB: Carbapenem-resistant *A. baumannii*; MSSA: Persistent methicillin-sensitive *S. aureus*; PKI: Orosthetic knee infection; UTI: Urinary tract infection; CRP: C-reactive protein; WBC: white blood cells.

**Table 3:** A compilation of clinical studies that have conducted safety monitoring of phage therapy against MDR bacteria.

Studies	Phage/s		Against	Phage Distribution	Normal Imaging/Lab Assessment (e.g., X-rays, Liver Function)	Presence of Abnormal (Increase or Decrease) of		Adverse Events
	Phage/s (Administration Route)	Endotoxin within Acceptable Range				Cell Infiltration/Cytokine Production	Antibodies Production	
Leitner et al., 2020 [134]	Pyo Phage (Local)	Yes, <0.5 EU/mL	MDR uropathogens (*Enterococcus* spp., *E. coli*, *Proteus mirabilis*, *P. aeruginosa*, *Staphylococcus* spp., and *Streptococcus* spp.) in UTI		Yes			Sudden onset of fever (>38 °C)
Fabijan et al., 2020 [135]	AB-SA01 (IV)		MRSA in infection	Detected in blood up to 12 h after dosing, and valve tissues at day 14 of phage treatment.	Yes	Not present. (Decline in inflammatory markers)		No adverse reactions (fever, tachycardia, hypotension, diarrhea, or abdominal pain and the development of renal or hepatic dysfunction)
Wright et al., 2009 [136]	Biophage-PA. (Local)		Antibiotic-resistant *P. aeruginosa* in chronic otitis	Detected in ear during post- treatment		Present in some patients. Assessed via VAS		None

Gray columns indicate that the respective aspects were not reported in the study. MDR: Multidrug resistant; MRSA: Methicillin resistant *S. aureus*: UTI: Urinary tract infection; JAK-STAT: Janus kinase/signal transducers and activators of transcription; VAS: Visual Analogue Scale.

## Data Availability

Not applicable.

## References

[1] Sweeney M.T., Lubbers B.V., Schwarz S., Watts J.L. (2018). Applying definitions for multidrug resistance, extensive drug resistance and pandrug resistance to clinically significant livestock and companion animal bacterial pathogens. J. Antimicrob. Chemother..

[2] Magiorakos A.P., Srinivasan A., Carey R.B., Carmeli Y., Falagas M.E., Giske C.G., Harbarth S., Hindler J.F., Kahlmeter G., Olsson-Liljequist B. (2012). Multidrug-resistant, extensively drug-resistant and pandrug-resistant bacteria: An international expert proposal for interim standard definitions for acquired resistance. Clin. Microbiol. Infect..

[3] De Tejada G.M., Heinbockel L., Ferrer-Espada R., Heine H., Alexander C., Bárcena-Varela S., Goldmann T., Correa W., Wiesmüller K., Gisch N. (2015). Lipoproteins/peptides are sepsis-inducing toxins from bacteria that can be neutralized by synthetic anti-endotoxin peptides. Sci. Rep..

[4] Ling H., Lou X., Luo Q., He Z., Sun M., Sun J. (2022). Recent advances in bacteriophage-based therapeutics: Insight into the post-antibiotic era. Acta Pharm. Sin. B.

[5] Ferry T., Kolenda C., Batailler C., Gustave C.A., Lustig S., Malatray M., Fevre C., Josse J., Petitjean C., Chidiac C. (2020). Phage Therapy as Adjuvant to Conservative Surgery and Antibiotics to Salvage Patients with Relapsing *S. aureus* Prosthetic Knee Infection. Front. Med..

[6] Fong S.A., Drilling A.J., Ooi M.L., Paramasivan S., Finnie J.W., Morales S., Psaltis A.J., Vreugde S., Wormald P. (2019). Safety and efficacy of a bacteriophage cocktail in an in vivo model of *Pseudomonas aeruginosa* sinusitis. Transl. Res. J. Lab. Clin. Med..

[7] Lebeaux D., Merabishvili M., Caudron E., Lannoy D., Van Simaey L., Duyvejonck H., Guillemain R., Thumerelle C., Podglajen I., Compain F. (2021). A Case of Phage Therapy against Pandrug-Resistant *Achromobacter xylosoxidans* in a 12-Year-Old Lung-Transplanted Cystic Fibrosis Patient. Viruses.

[8] Yin S., Huang G., Zhang Y., Jiang B., Yang Z., Dong Z., You B., Yuan Z., Hu F., Zhao Y. (2017). Phage Abp1 Rescues Human Cells and Mice from Infection by Pan-Drug Resistant *Acinetobacter Baumannii*. Cell. Physiol. Biochem..

[9] Dedrick R.M., Guerrero-Bustamante C.A., Garlena R.A., Russell D.A., Ford K., Harris K., Gilmour K.C., Soothill J., Jacobs-Sera D., Schooley R.T. (2019). Engineered bacteriophages for treatment of a patient with a disseminated drug resistant *Mycobacterium abscessus*. Nat. Med..

[10] Doub J.B., Ng V.Y., Johnson A.J., Slomka M., Fackler J., Horne B., Brownstein M.J., Henry M., Malagon F., Biswas B. (2020). Salvage Bacteriophage Therapy for a Chronic MRSA Prosthetic Joint Infection. Antibiotics.

[11] LaVergne S., Hamilton T., Biswas B., Kumaraswamy M., Schooley R.T., Wooten D. (2018). Phage Therapy for a Multidrug-Resistant *Acinetobacter baumannii* Craniectomy Site Infection. Open Forum Infect. Dis..

[12] Lindford A., Kiuru V., Anttila V.J., Vuola J. (2015). Successful eradication of multidrug resistant *acinetobacter* in the Helsinki burn centre. J. Burn. Care Res..

[13] Karumathil D.P., Nair M.S., Gaffney J., Kollanoor-Johny A., Venkitanarayanan K. (2018). Trans-Cinnamaldehyde and eugenol increase *Acinetobacter baumannii* sensitivity to beta-lactam antibiotics. Front. Microbiol..

[14] Tsuji B.T., Pogue J.M., Zavascki A.P., Paul M., Daikos G.L., Forrest A., Giacobbe D.R., Viscoli C., Giamarellou H., Karaiskos I. (2019). International Consensus Guidelines for the Optimal Use of the Polymyxins: Endorsed by the American College of Clinical Pharmacy (ACCP), European Society of Clinical Microbiology and Infectious Diseases (ESCMID), Infectious Diseases Society of America (IDSA), International Society for Anti-infective Pharmacology (ISAP), Society of Critical Care Medicine (SCCM), and Society of Infectious Diseases Pharmacists (SIDP). Pharmacother. J. Hum. Pharmacol. Drug Ther..

[15] Yang Z., Shi Y., Zhang C., Luo X., Chen Y., Peng Y., Gong Y. (2019). Lytic Bacteriophage Screening Strategies for Multidrug-Resistant Bloodstream Infections in a Burn Intensive Care Unit. Med. Sci. Monit. Int. Med. J. Exp. Clin. Res..

[16] *WHO Publishes List of Bacteria for Which New Antibiotics Are Urgently Needed*; World Health Organization: Geneva, Switzerland, 2017. https://www.who.int/news/item/27-02-2017-who-publishes-list-of-bacteria-for-which-new-antibiotics-are-urgently-needed.

[17] Hendrix R.W., Smith M.C.M., Burns R.N., Ford M.E., Hatfull G.F. (1999). Evolutionary relationships among diverse bacteriophages and prophages: All the world’s a phage. Proc. Natl. Acad. Sci. USA.

[18] Kim S.H., Adeyemi D.E., Park M.K. (2021). Characterization of a new and efficient polyvalent phage infecting *E. coli* o157:H7, *Salmonella* spp., and *Shigella sonnei*. Microorganisms.

[19] Hudson J.A., Billington C., Wilson T., On S.L.W. (2015). Effect of phage and host concentration on the inactivation of *Escherichia coli* O157:H7 on cooked and raw beef. Food Sci. Technol. Int. = Cienc. Y Tecnol. De Los Aliment. Int..

[20] Duc H.M., Son H.M., Yi H.P.S., Sato J., Ngan P.H., Masuda Y., Honjoh K., Miyamoto T. (2020). Isolation, characterization and application of a polyvalent phage capable of controlling *Salmonella* and *Escherichia coli* O157:H7 in different food matrices. Food Res. Int..

[21] Sui B., Han L., Ren H., Liu W., Zhang C. (2021). A Novel Polyvalent Bacteriophage vB_EcoM_swi3 Infects Pathogenic *Escherichia coli* and *Salmonella enteritidis*. Front. Microbiol..

[22] Sieiro C., Areal-Hermida L., Pichardo-Gallardo Á., Almuiña-González R., De Miguel T., Sánchez S., Sánchez-Pérez Á., Villa T.G. (2020). A Hundred Years of Bacteriophages: Can Phages Replace Antibiotics in Agriculture and Aquaculture?. Antibiotics.

[23] d’Herelle F. (1931). An Address on Bacteriophagy and Recovery from Infectious Diseases. Can. Med. Assoc. J..

[24] d’Herelle F. (1931). Annual Graduate Fortnight. Medical and Surgical Aspects of Acute Bacterial Infections, October 20 to 31, 1930: Bacteriophage as a Treatment in Acute Medical and Surgical Infections. Bull. N. Y. Acad. Med..

[25] Pirnay J.P., De Vos D., Verbeken G., Merabishvili M., Chanishvili N., Vaneechoutte M., Zizi M., Laire G., Lavigne R., Huys I. (2011). The phage therapy paradigm: Prêt-à-porter or sur-mesure?. Pharm. Res..

[26] Arumugam S.N., Rudraradhya A.C., Sadagopan S., Sukumaran S., Sambasivam G., Ramesh N. (2018). Analysis of susceptibility patterns of *pseudomonas aeruginosa* and Isolation, Characterization of lytic bacteriophages targeting multi drug resistant *Pseudomonas aeruginosa*. Biomed. Pharmacol. J..

[27] Nepal R., Houtak G., Karki S., Dhungana G., Vreugde S., Malla R. (2022). Genomic characterization of three bacteriophages targeting multidrug resistant clinical isolates of *Escherichia*, *Klebsiella* and *Salmonella*. Arch. Microbiol..

[28] Tao C., Yi Z., Zhang Y., Wang Y., Zhu H., Afayibo D.J.A., Li T., Tian M., Qi J., Ding C. (2021). Characterization of a Broad-Host-Range Lytic Phage SHWT1 Against Multidrug-Resistant *Salmonella* and Evaluation of Its Therapeutic Efficacy in vitro and in vivo. Front. Vet. Sci..

[29] Deng L.Y., Yang Z.C., Gong Y.L., Huang G.T., Yin S.P., Jiang B., Peng Y.Z. (2016). Therapeutic effect of phages on extensively drug-resistant *Acinetobacter baumannii*-induced sepsis in mice. Chin. J. Burn..

[30] Schooley R.T., Biswas B., Gill J.J., Hernandez-Morales A., Lancaster J., Lessor L., Barr J.J., Reed S.L., Rohwer F., Benler S. (2017). Development and Use of Personalized Bacteriophage-Based Therapeutic Cocktails to Treat a Patient with a Disseminated Resistant *Acinetobacter baumannii* Infection. Antimicrob. Agents Chemother..

[31] Aslam S., Courtwright A.M., Koval C., Lehman S.M., Morales S., Furr C.L.L., Rosas F., Brownstein M.J., Fackler J.R., Sisson B.M. (2019). Early clinical experience of bacteriophage therapy in 3 lung transplant recipients. Am. J. Transplantation.

[32] Rubalskii E., Ruemke S., Salmoukas C., Boyle E.C., Warnecke G., Tudorache I., Shrestha M., Schmitto J.D., Martens A., Rojas S.V. (2020). Bacteriophage Therapy for Critical Infections Related to Cardiothoracic Surgery. Antibiotics.

[33] Wu N., Dai J., Guo M., Li J., Zhou X., Li F., Gao Y., Qu H., Lu H., Jin J. (2021). Pre-optimized phage therapy on secondary *Acinetobacter baumannii* infection in four critical COVID-19 patients. Emerg. Microbes Infect..

[34] Nick J.A., Dedrick R.M., Gray A.L., Vladar E.K., Smith B.E., Freeman K.G., Malcolm K.C., Epperson L.E., Hasan N.A., Hendrix J. (2022). Host and pathogen response to bacteriophage engineered against *Mycobacterium abscessus* lung infection. Cell.

[35] Martin M.J., Thottathil S.E., Newman T.B. (2015). Antibiotics overuse in animal agriculture: A call to action for health care providers. Am. J. Public Health.

[36] Muloi D., Ward M.J., Pedersen A.B., Fèvre E.M., Woolhouse M.E.J., Van Bunnik B.A.D. (2018). Are Food Animals Responsible for Transfer of Antimicrobial-Resistant *Escherichia coli* or Their Resistance Determinants to Human Populations? A Systematic Review. Foodborne Pathog. Dis..

[37] Ahmed H., Zolfo M., Williams A., Ashubwe-Jalemba J., Tweya H., Adeapena W., Labi A., Adomako L.A.B., Addico G.N.D., Banu R.A. (2022). Antibiotic-Resistant Bacteria in Drinking Water from the Greater Accra Region, Ghana: A Cross-Sectional Study, December 2021–March 2022. Int. J. Environ. Res. Public Health.

[38] Bamigboye C.O., Amao J.A., Ayodele T.A., Adebayo A.S., Ogunleke J.D., Abass T.B., Oyedare T.A., Adetutu T.J., Adeeyo A.O., Oyedemi A.A. (2020). An appraisal of the drinking water quality of groundwater sources in Ogbomoso, Oyo state, Nigeria. Groundw. Sustain. Dev..

[39] Odonkor S.T., Simpson S.V., Morales Medina W.R., Fahrenfeld N.L. (2022). Antibiotic-Resistant Bacteria and Resistance Genes in Isolates from Ghanaian Drinking Water Sources. J. Environ. Public Health.

[40] Adesoji A.T., Onuh J.P., Musa A.O., Akinrosoye P.F. (2019). Bacteriological qualities and antibiogram studies of bacteria from “suya” and smoked fish (*Clarias gariepinus*) in Dutsin-Ma, Katsina State, Nigeria. Pan Afr. Med. J..

[41] Lauteri C., Festino A.R., Conter M., Vergara A. (2022). Prevalence and antimicrobial resistance profile in *Salmonella* spp. isolates from swine food chain. Ital. J. Food Saf..

[42] Rau R.B., Ribeiro A.R., Dos Santos A., Barth A.L. (2021). Antimicrobial resistance of *Salmonella* from poultry meat in Brazil: Results of a nationwide survey. Epidemiol. Infect..

[43] Andreoletti O., Lau Baggesen D., Bolton D., Butaye P., Cook P., Davies R., Escámez P.S.F., Griffin J., Hald T., Havelaar A. (2013). Scientific Opinion on the risk posed by pathogens in food of non-animal origin. Part 1 (outbreak data analysis and risk ranking of food/pathogen combinations). EFSA J..

[44] Rahman M., Alam M.U., Luies S.K., Kamal A., Ferdous S., Lin A., Sharior F., Khan R., Rahman Z., Parvez S.M. (2022). Contamination of fresh produce with antibiotic-resistant bacteria and associated risks to human health: A scoping review. Int. J. Environ. Res. Public Health.

[45] Verma P., Saharan V.V., Nimesh S., Singh A.P. (2018). Phenotypic and virulence traits of *Escherichia coli* and *Salmonella* strains isolated from vegetables and fruits from India. J. Appl. Microbiol..

[46] Olowe O.A., Adefioye O.J., Ajayeoba T.A., Schiebel J., Weinreich J., Ali A., Burdukiewicz M., Rödiger S., Schierack P. (2019). Phylogenetic grouping and biofilm formation of multidrug resistant *Escherichia coli* isolates from humans, animals and food products in South-West Nigeria. Sci. Afr..

[47] Naghizadeh M., Karimi Torshizi M.A., Rahimi S., Engberg R.M., Sørensen Dalgaard T. (2019). Effect of serum anti-phage activity on colibacillosis control by repeated phage therapy in broilers. Vet. Microbiol..

[48] Guo M., Gao Y., Xue Y., Liu Y., Zeng X., Cheng Y., Ma J., Wang H., Sun J., Wang Z. (2021). Bacteriophage Cocktails Protect Dairy Cows Against Mastitis Caused By Drug Resistant *Escherichia coli* Infection. Front. Cell. Infect. Microbiol..

[49] Tolen T.N., Xie Y., Hairgrove T.B., Gill J.J., Matthew Taylor T. (2018). Evaluation of commercial prototype bacteriophage intervention designed for reducing O157 and non-O157 Shiga-toxigenic *Escherichia coli* (STEC) on beef cattle hide. Foods.

[50] Verstappen K.M., Tulinski P., Duim B., Fluit A.C., Carney J., Van Nes A., Wagenaar J.A. (2016). The Effectiveness of Bacteriophages against Methicillin-Resistant *Staphylococcus aureus* ST398 Nasal Colonization in Pigs. PLoS ONE.

[51] Thanki A.M., Brown N., Millard A.D., Clokie M.R.J. (2019). Genomic characterization of jumbo *Salmonella* phages that effectively target United Kingdom pig-associated *Salmonella* serotypes. Front. Microbiol..

[52] Chen L., Fan J., Yan T., Liu Q., Yuan S., Zhang H., Yang J., Deng D., Huang S., Ma Y. (2019). Isolation and Characterization of Specific Phages to Prepare a Cocktail Preventing *Vibrio* sp. Va-F3 Infections in Shrimp (*Litopenaeus vannamei*). Front. Microbiol..

[53] Le T.S., Southgate P.C., O’connor W., Vu S.V., İpek Kurtböke D. (2020). Application of Bacteriophages to Control *Vibrio alginolyticus* Contamination in Oyster (*Saccostrea glomerata*) Larvae. Antibiotics.

[54] Wang X., Wei Z., Yang K., Wang J., Jousset A., Xu Y., Shen Q., Friman V.P. (2019). Phage combination therapies for bacterial wilt disease in tomato. Nat. Biotechnol..

[55] Carstens A.B., Djurhuus A.M., Kot W., Hansen L.H. (2019). A novel six-phage cocktail reduces *Pectobacterium atrosepticum* soft rot infection in potato tubers under simulated storage conditions. FEMS Microbiol. Lett..

[56] Zaczek-Moczydłowska M.A., Young G.K., Trudgett J., Plahe C., Fleming C.C., Campbell K., O’Hanlon R. (2020). Phage cocktail containing Podoviridae and Myoviridae bacteriophages inhibits the growth of *Pectobacterium* spp. under in vitro and in vivo conditions. PLoS ONE.

[57] Papaianni M., Paris D., Woo S.L., Fulgione A., Rigano M.M., Parrilli E., Tutino M.L., Marra R., Manganiello G., Casillo A. (2020). Plant Dynamic Metabolic Response to Bacteriophage Treatment After *Xanthomonas campestris* pv. campestris Infection. Front. Microbiol..

[58] Vikram A., Tokman J.I., Woolston J., Sulakvelidze A. (2020). Phage Biocontrol Improves Food Safety by Significantly Reducing the Level and Prevalence of *Escherichia coli* O157:H7 in Various Foods. J. Food Prot..

[59] Xu J., Zhang R., Yu X., Zhang X., Liu G., Liu X. (2021). Molecular Characteristics of Novel Phage vB_ShiP-A7 Infecting Multidrug-Resistant *Shigella flexneri* and *Escherichia coli*, and Its Bactericidal Effect in vitro and in vivo. Front. Microbiol..

[60] Rangasamy K., Murugan A., Devarajan N., Parray J.A. (2017). Emergence of multi drug resistance among soil bacteria exposing to insecticides. Microb. Pathog..

[61] Mahdiyah D., Farida H., Riwanto I., Mustofa M., Wahjono H., Laksana Nugroho T., Reki W. (2020). Screening of Indonesian peat soil bacteria producing antimicrobial compounds. Saudi J. Biol. Sci..

[62] Mafiz A.I., He Y., Zhang W., Zhang Y. (2021). Soil Bacteria in Urban Community Gardens Have the Potential to Disseminate Antimicrobial Resistance Through Horizontal Gene Transfer. Front. Microbiol..

[63] Yahya M., Hmaied F., Jebri S., Jofre J., Hamdi M. (2015). Bacteriophages as indicators of human and animal faecal contamination in raw and treated wastewaters from Tunisia. J. Appl. Microbiol..

[64] Calero-Cáceres W., Balcázar J.L. (2019). Antibiotic resistance genes in bacteriophages from diverse marine habitats. Sci. Total Environ..

[65] Lekunberri I., Subirats J., Borrego C.M., Balcázar J.L. (2017). Exploring the contribution of bacteriophages to antibiotic resistance. Environ. Pollut..

[66] Loc-Carrillo C., Abedon S.T. (2011). Pros and cons of phage therapy. Bacteriophage.

[67] McCallin S., Alam Sarker S., Barretto C., Sultana S., Berger B., Huq S., Krause L., Bibiloni R., Schmitt B., Reuteler G. (2013). Safety analysis of a Russian phage cocktail: From MetaGenomic analysis to oral application in healthy human subjects. Virology.

[68] Febvre H.P., Rao S., Gindin M., Goodwin N.D.M., Finer E., Vivanco J.S., Manter D.K., Wallace T.C., Weir T.L. (2019). PHAGE study: Effects of supplemental bacteriophage intake on inflammation and gut microbiota in healthy adults. Nutrients.

[69] Galtier M., De Sordi L., Maura D., Arachchi H., Volant S., Dillies M.A., Debarbieux L. (2016). Bacteriophages to reduce gut carriage of antibiotic resistant uropathogens with low impact on microbiota composition. Environ. Microbiol..

[70] Grubb D.S., Wrigley S.D., Freedman K.E., Wei Y., Vazquez A.R., Trotter R.E., Wallace T.C., Johnson S.A., Weir T.L. (2020). PHAGE-2 Study: Supplemental Bacteriophages Extend *Bifidobacterium animalis* subsp. lactis BL04 Benefits on Gut Health and Microbiota in Healthy Adults. Nutrients.

[71] Gunathilaka G.U., Tahlan V., Mafiz A.I., Polur M., Zhang Y. (2017). Phages in urban wastewater have the potential to disseminate antibiotic resistance. Int. J. Antimicrob. Agents.

[72] Liu J., Liu P., Feng F., Zhang J., Li F., Wang M., Sun Y. (2020). Evaluation of Potential ARG Packaging by Two Environmental T7-Like Phage during Phage-Host Interaction. Viruses.

[73] Zhang Y., Guo Y., Qiu T., Gao M., Wang X. (2022). Bacteriophages: Underestimated vehicles of antibiotic resistance genes in the soil. Front. Microbiol..

[74] Frazão N., Sousa A., Lässig M., Gordo I. (2019). Horizontal gene transfer overrides mutation in *Escherichia coli* colonizing the mammalian gut. Proc. Natl. Acad. Sci. USA.

[75] Fernández-Orth D., Miró E., Brown-Jaque M., Rodríguez-Rubio L., Espinal P., Rodriguez-Navarro J., González-López J.J., Muniesa M., Navarro F. (2019). Faecal phageome of healthy individuals: Presence of antibiotic resistance genes and variations caused by ciprofloxacin treatment. J. Antimicrob. Chemother..

[76] Enault F., Briet A., Bouteille L., Roux S., Sullivan M.B., Petit M.A. (2016). Phages rarely encode antibiotic resistance genes: A cautionary tale for virome analyses. ISME J..

[77] Chan B.K., Abedon S.T., Loc-Carrillo C. (2013). Phage cocktails and the future of phage therapy. Future Microbiol..

[78] Ujmajuridze A., Chanishvili N., Goderdzishvili M., Leitner L., Mehnert U., Chkhotua A., Kessler T.M., Sybesma W. (2018). Adapted bacteriophages for treating urinary tract infections. Front. Microbiol..

[79] (2013). Infusion Related Reactions Guidance 2013.

[80] Moghadam M.T., Amirmozafari N., Shariati A., Hallajzadeh M., Mirkalantari S., Khoshbayan A., Jazi F.M. (2020). How phages overcome the challenges of drug resistant bacteria in clinical infections. Infect. Drug Resist..

[81] Van Belleghem J.D., Clement F., Merabishvili M., Lavigne R., Vaneechoutte M. (2017). Pro- and anti-inflammatory responses of peripheral blood mononuclear cells induced by *Staphylococcus aureus* and *Pseudomonas aeruginosa* phages. Sci. Rep..

[82] Zhang L., Hou X., Sun L., He T., Wei R., Pang M., Wang R. (2018). Corrigendum: *Staphylococcus aureus* Bacteriophage Suppresses LPS-Induced Inflammation in MAC-T Bovine Mammary Epithelial Cells. Front. Microbiol..

[83] Dufour N., Delattre R., Chevallereau A., Ricard J.D., Debarbieux L. (2019). Phage Therapy of Pneumonia Is Not Associated with an Overstimulation of the Inflammatory Response Compared to Antibiotic Treatment in Mice. Antimicrob. Agents Chemother..

[84] Majewska J., Kaźmierczak Z., Lahutta K., Lecion D., Szymczak A., Miernikiewicz P., Drapała J., Harhala M., Marek-Bukowiec K., Jędruchniewicz N. (2019). Induction of Phage-Specific Antibodies by Two Therapeutic *Staphylococcal bacteriophages* Administered per os. Front. Immunol..

[85] Jun J.W., Shin T.H., Kim J.H., Shin S.P., Han J.E., Heo G.J., De Zoysa M., Shin G.W., Chai J.Y., Park S.C. (2014). Bacteriophage Therapy of a *Vibrio parahaemolyticus* Infection Caused by a Multiple-Antibiotic–Resistant O3:K6 Pandemic Clinical Strain. J. Infect. Dis..

[86] Sunagar R., Patil S.A., Chandrakanth R.K. (2010). Bacteriophage therapy for *Staphylococcus aureus* bacteremia in streptozotocin-induced diabetic mice. Res. Microbiol..

[87] Gainey A.B., Burch A.K., Brownstein M.J., Brown D.E., Fackler J., Horne B., Biswas B., Bivens B.N., Malagon F., Daniels R. (2020). Combining bacteriophages with cefiderocol and meropenem/vaborbactam to treat a pan-drug resistant *Achromobacter* species infection in a pediatric cystic fibrosis patient. Pediatr. Pulmonol..

[88] Moghadam M.T., Khoshbayan A., Chegini Z., Farahani I., Shariati A. (2020). Bacteriophages, a New Therapeutic Solution for Inhibiting Multidrug-Resistant Bacteria Causing Wound Infection: Lesson from Animal Models and Clinical Trials. Drug Des. Dev. Ther..

[89] Park K., Cha K.E., Myung H. (2014). Observation of inflammatory responses in mice orally fed with bacteriophage T7. J. Appl. Microbiol..

[90] (2018). Setting Endotoxin Acceptance Criteria for Biologics Intravenous (IV) and Subcutaneous (SC) Mono- and Combination Therapies| American Pharmaceutical Review—The Review of American Pharmaceutical Business & Technology. American Pharmaceutical Review. https://www.americanpharmaceuticalreview.com/Featured-Articles/353671-Setting-Endotoxin-Acceptance-Criteria-for-Biologics-Intravenous-IV-and-Subcutaneous-SC-Mono-and-Combination-Therapies/.

[91] Cooper C.J., Mirzaei M.K., Nilsson A.S. (2016). Adapting drug approval pathways for bacteriophage-based therapeutics. Front. Microbiol..

[92] Bao H., Zhang H., Zhou Y., Zhu S., Pang M., Shahin K., Shahin K., Olaniran A., Schmidt S., Wang R. (2020). Transient carriage and low-level colonization of orally administrated lytic and temperate phages in the gut of mice. Food Prod. Process. Nutr..

[93] Cano E.J., Caflisch K.M., Bollyky P.L., Van Belleghem J.D., Patel R., Fackler J., Brownstein M.J., Horne B., Biswas B., Henry M. (2021). Phage Therapy for Limb-threatening Prosthetic Knee *Klebsiella pneumoniae* Infection: Case Report and In Vitro Characterization of Anti-biofilm Activity. Clin. Infect. Dis..

[94] Jault P., Leclerc T., Jennes S., Pirnay J.P., Que Y.A., Resch G., Rousseau A.F., Ravat F., Carsin H., Le Floch R. (2019). Efficacy and tolerability of a cocktail of bacteriophages to treat burn wounds infected by *Pseudomonas aeruginosa* (PhagoBurn): A randomised, controlled, double-blind phase 1/2 trial. Lancet Infect. Dis..

[95] Wang Y., Han S. (2022). Bacterial DNA involvement in carcinogenesis. Front. Cell. Infect. Microbiol..

[96] Gilbey T., Ho J., Cooley L.A., Petrovic Fabijan A., Iredell J.R. (2019). Adjunctive bacteriophage therapy for prosthetic valve endocarditis due to *Staphylococcus aureus*. Med. J. Aust..

[97] Janik E., Ceremuga M., Bijak J.S., Bijak M. (2019). Biological Toxins as the Potential Tools for Bioterrorism. Int. J. Mol. Sci..

[98] Kissner T.L., Cisney E.D., Ulrich R.G., Fernandez S., Saikh K.U. (2010). *Staphylococcal* enterotoxin A induction of pro-inflammatory cytokines and lethality in mice is primarily dependent on MyD88. Immunology.

[99] Otto M. (2014). *Staphylococcus aureus* toxins. Curr. Opin. Microbiol..

[100] Ho S.S., Michalek S.M., Nahm M.H. (2008). Lipoteichoic Acid Is Important in Innate Immune Responses to Gram-Positive Bacteria. Infect. Immun..

[101] Berube B.J., Wardenburg J.B. (2013). *Staphylococcus aureus* α-Toxin: Nearly a Century of Intrigue. Toxins.

[102] Szentirmai É., Massie A.R., Kapás L. (2021). Lipoteichoic acid, a cell wall component of Gram-positive bacteria, induces sleep and fever and suppresses feeding. Brain Behav. Immun..

[103] Guerin K., Choi V., Aranda J., Demirs J., Li H., Yang J., Nguyen N., Bottega S., Jaffee B., Dryja T. (2015). Residual Cesium Chloride in AAV Vectors Purified by CsCl Gradient Centrifugation Does Not Cause Obvious Inflammation or Retinal Degeneration in C57Bl6/J Mice Following Subretinal Injection. Mol. Ther..

[104] Hietala V., Horsma-Heikkinen J., Carron A., Skurnik M., Kiljunen S. (2019). The Removal of Endo- and Enterotoxins from Bacteriophage Preparations. Front. Microbiol..

[105] Chaudhary K. (2018). BacteRiophage EXclusion (BREX): A novel anti-phage mechanism in the arsenal of bacterial defense system. J. Cell. Physiol..

[106] Liu M., Deora R., Doulatov S.R., Gingery M., Eiserling F.A., Preston A., Maskell D.J., Simons R.W., Cotter P.A., Parkhill J. (2002). Reverse transcriptase-mediated tropism switching in *Bordetella* bacteriophage. Science.

[107] Fineran P.C., Blower T.R., Foulds I.J., Humphreys D.P., Lilley K.S., Salmond G.P.C. (2009). The phage abortive infection system, ToxIN, functions as a protein-RNA toxin-antitoxin pair. Proc. Natl. Acad. Sci. USA.

[108] Blower T.R., Fineran P.C., Johnson M.J., Toth I.K., Humphreys D.P., Salmond G.P.C. (2009). Mutagenesis and functional characterization of the RNA and protein components of the toxIN abortive infection and toxin-antitoxin locus of Erwinia. J. Bacteriol..

[109] Larsson D.G.J. (2014). Antibiotics in the environment. Upsala J. Med. Sci..

[110] Litt P.K., Jaroni D. (2017). Isolation and Physiomorphological Characterization of *Escherichia coli* O157:H7-Infecting Bacteriophages Recovered from Beef Cattle Operations. Int. J. Microbiol..

[111] Meaden S., Koskella B. (2013). Exploring the risks of phage application in the environment. Front. Microbiol..

[112] Drilling A.J., Ooi M.L., Miljkovic D., James C., Speck P., Vreugde S., Clark J., Wormald P.J. (2017). Long-Term Safety of Topical Bacteriophage Application to the Frontal Sinus Region. Front. Cell. Infect. Microbiol..

[113] Liu D., Van Belleghem J.D., de Vries C.R., Burgener E., Chen Q., Manasherob R., Aronson J.R., Amanatullah D.F., Tamma P.D., Suh G.A. (2021). The safety and toxicity of phage therapy: A review of animal and clinical studies. Viruses.

[114] Onsea J., Soentjens P., Djebara S., Merabishvili M., Depypere M., Spriet I., De Munter P., Debaveye Y., Njis S., Vanderschot P. (2019). Bacteriophage Application for Difficult-To-Treat Musculoskeletal Infections: Development of a Standardized Multidisciplinary Treatment Protocol. Viruses.

[115] Khatami A., Lin R.C.Y., Petrovic-Fabijan A., Alkalay-Oren S., Almuzam S., Britton P.N., Brownstein M.J., Dao Q., Fackler J., Hazan R. (2021). Bacterial lysis, autophagy and innate immune responses during adjunctive phage therapy in a child. EMBO Mol. Med..

[116] Aslam S., Lampley E., Wooten D., Karris M., Benson C., Strathdee S., Schooley R.T. (2020). Lessons Learned From the First 10 Consecutive Cases of Intravenous Bacteriophage Therapy to Treat Multidrug-Resistant Bacterial Infections at a Single Center in the United States. Open Forum Infect. Dis..

[117] Bao J., Wu N., Zeng Y., Chen L., Li L., Yang L., Zhang Y., Guo M., Li L., Li J. (2020). Non-active antibiotic and bacteriophage synergism to successfully treat recurrent urinary tract infection caused by extensively drug-resistant *Klebsiella pneumoniae*. Emerg. Microbes Infect..

[118] Eskenazi A., Lood C., Wubbolts J., Hites M., Balarjishvili N., Leshkasheli L., Askilashvili L., Kvachadze L., van Noort V., Wagemans J. (2022). Combination of pre-adapted bacteriophage therapy and antibiotics for treatment of fracture-related infection due to pandrug-resistant *Klebsiella pneumoniae*. Nat. Commun..

[119] Johri A.V., Johri P., Hoyle N., Pipia L., Nadareishvili L., Nizharadze D. (2021). Case Report: Chronic Bacterial Prostatitis Treated With Phage Therapy After Multiple Failed Antibiotic Treatments. Front. Pharmacol..

[120] Ramirez-Sanchez C., Gonzales F., Buckley M., Biswas B., Henry M., Deschenes M.V., Horne B., Fackler J., Brownstein M.J., Schooley R.T. (2021). Successful Treatment of *Staphylococcus aureus* Prosthetic Joint Infection with Bacteriophage Therapy. Viruses.

[121] Rostkowska O.M., Międzybrodzki R., Miszewska-Szyszkowska D., Górski A., Durlik M. (2021). Treatment of recurrent urinary tract infections in a 60-year-old kidney transplant recipient. The use of phage therapy. Transpl. Infect. Dis..

[122] Corbellino M., Kieffer N., Kutateladze M., Balarjishvili N., Leshkasheli L., Askilashvili L., Tsertsvadze G., Rimoldi S.G., Nizharadze D., Hoyle N. (2020). Eradication of a Multidrug-Resistant, Carbapenemase-Producing *Klebsiella pneumoniae* Isolate Following Oral and Intra-rectal Therapy With a Custom Made, Lytic Bacteriophage Preparation. Clin. Infect. Dis..

[123] Nir-Paz R., Gelman D., Khouri A., Sisson B.M., Fackler J., Alkalay-Oren S., Khalifa L., Rimon A., Yerushalmy O., Bader R. (2019). Successful Treatment of Antibiotic-resistant, Poly-microbial Bone Infection With Bacteriophages and Antibiotics Combination. Clin. Infect. Dis..

[124] Tkhilaishvili T., Winkler T., Müller M., Perka C., Trampuz A. (2020). Bacteriophages as Adjuvant to Antibiotics for the Treatment of Periprosthetic Joint Infection Caused by Multidrug-Resistant *Pseudomonas aeruginosa*. Antimicrob. Agents Chemother..

[125] Maddocks S., Fabijan A.P., Ho J., Lin R.C.Y., Ben Zakour N.L., Dugan C., Kliman I., Branston S., Morales S., Iredell J.R. (2019). Bacteriophage Therapy of Ventilator-associated Pneumonia and Empyema Caused by *Pseudomonas aeruginosa*. Am. J. Respir. Crit. Care Med..

[126] Law N., Logan C., Yung G., Furr C.L.L., Lehman S.M., Morales S., Rosas F., Gaidamaka A., Bilinsky I., Grint P. (2019). Successful adjunctive use of bacteriophage therapy for treatment of multidrug-resistant *Pseudomonas aeruginosa* infection in a cystic fibrosis patient. Infection.

[127] Kuipers S., Ruth M.M., Mientjes M., de Sévaux R.G.L., van Ingen J. (2020). A Dutch Case Report of Successful Treatment of Chronic Relapsing Urinary Tract Infection with Bacteriophages in a Renal Transplant Patient. Antimicrob. Agents Chemother..

[128] Aslam S., Pretorius V., Lehman S.M., Morales S., Schooley R.T. (2019). Novel bacteriophage therapy for treatment of left ventricular assist device infection. J. Heart Lung Transplantation.

[129] Ferry T., Boucher F., Fevre C., Perpoint T., Chateau J., Petitjean C., Josse J., Chidiac C., L’hostis G., Leboucher G. (2018). Innovations for the treatment of a complex bone and joint infection due to XDR *Pseudomonas aeruginosa* including local application of a selected cocktail of bacteriophages. J. Antimicrob. Chemother..

[130] Duplessis C., Biswas B., Hanisch B., Perkins M., Henry M., Quinones J., Wolfe D., Estrella L., Hamilton T. (2018). Refractory *Pseudomonas* Bacteremia in a 2-Year-Old Sterilized by Bacteriophage Therapy. J. Pediatr. Infect. Dis. Soc..

[131] Ferry T., Leboucher G., Fevre C., Herry Y., Conrad A., Josse J., Batailler C., Chidiac C., Medina M., Lustig S. (2018). Salvage Debridement, Antibiotics and Implant Retention (“DAIR”) With Local Injection of a Selected Cocktail of Bacteriophages: Is It an Option for an Elderly Patient With Relapsing *Staphylococcus aureus* Prosthetic-Joint Infection?. Open Forum Infect. Dis..

[132] Zhvania P., Hoyle N.S., Nadareishvili L., Nizharadze D., Kutateladze M. (2017). Phage therapy in a 16-year-old boy with netherton syndrome. Front. Med..

[133] Jennes S., Merabishvili M., Soentjens P., Pang K.W., Rose T., Keersebilck E., Soete O., François P., Teodorescu S., Verween G. (2017). Use of bacteriophages in the treatment of colistin-only-sensitive *Pseudomonas aeruginosa* septicaemia in a patient with acute kidney injury—A case report. Crit. Care.

[134] Leitner L., Ujmajuridze A., Chanishvili N., Goderdzishvili M., Chkonia I., Rigvava S., Chkhotua A., Changashvili G., McCallin S., Schneider M.O. (2021). Intravesical bacteriophages for treating urinary tract infections in patients undergoing transurethral resection of the prostate: A randomised, placebo-controlled, double-blind clinical trial. Lancet. Infect. Dis..

[135] Fabijan A., Lin R.C.Y., Ho J., Maddocks S., Zakour N.L., Iredell J.R., Westmead Bacteriophage Therapy Team (2020). Safety of bacteriophage therapy in severe *Staphylococcus aureus* infection. Nat. Microbiol..

[136] Wright A., Hawkins C.H., Änggård E.E., Harper D.R. (2009). A controlled clinical trial of a therapeutic bacteriophage preparation in chronic otitis due to antibiotic-resistant *Pseudomonas aeruginosa*; a preliminary report of efficacy. Clin. Otolaryngol..

[137] Tang S.-S., Kumar Biswas S., Tan W.S., Saha A.K., Leo B.-F. (2019). Efficacy and potential of phage therapy against multidrug resistant *Shigella* spp.. PeerJ.

[138] Kim M., Jo Y., Hwang Y.J., Hong H.W., Hong S.S., Park K., Myung H. (2018). Phage-Antibiotic Synergy via Delayed Lysis. Appl. Environ. Microbiol..

[139] Uchiyama J., Shigehisa R., Nasukawa T., Mizukami K., Takemura-Uchiyama I., Ujihara T., Murakami H., Imanishi I., Nishifuji K., Sakaguchi M. (2018). Piperacillin and ceftazidime produce the strongest synergistic phage–antibiotic effect in *Pseudomonas aeruginosa*. Arch. Virol..

[140] Han M.L., Nang S.C., Lin Y.W., Zhu Y., Yu H.H., Wickremasinghe H., Barlow C.K., Creek D.J., Crawford S., Rao G. (2022). Comparative metabolomics revealed key pathways associated with the synergistic killing of multidrug-resistant *Klebsiella pneumoniae* by a bacteriophage-polymyxin combination. Comput. Struct. Biotechnol. J..

[141] Zuo P., Yu P., Alvarez P.J.J. (2021). Aminoglycosides Antagonize Bacteriophage Proliferation, Attenuating Phage Suppression of Bacterial Growth, Biofilm Formation, and Antibiotic Resistance. Appl. Environ. Microbiol..

